# Baseline platelet-to-lymphocyte ratio is associated with severe immune effector cell-associated toxicities in diffuse large B-cell lymphoma patients receiving anti-CD19 CAR T-cell therapy

**DOI:** 10.3389/fimmu.2026.1731711

**Published:** 2026-03-24

**Authors:** Changgon Kim, Kunye Kwak, Ka-Won Kang, Yong Park, Byung Soo Kim, Yoon Seok Choi

**Affiliations:** 1 Division of Hematology and Oncology, Department of Internal Medicine, Korea University College of Medicine, Seoul, Republic of Korea; 2 Department of Hematology, Catholic Hematology Hospital, Seoul St. Mary’s Hospital, College of Medicine, The Catholic University of Korea, Seoul, Republic of Korea

**Keywords:** diffuse large B-cell lymphoma, chimeric antigen receptor T-cell therapy, cytokine release syndrome, immune effector cell-associated neurotoxicity syndrome, platelet-to-lymphocyte ratio

## Abstract

**Introduction:**

Anti-CD19 chimeric antigen receptor (CAR) T-cell therapy can induce durable remissions in patients with relapsed or refractory diffuse large B-cell lymphoma (DLBCL); however, severe immune effector cell–associated adverse events, including cytokine release syndrome (CRS) and neurotoxicity, remain major clinical challenges. We investigated whether the platelet-to-lymphocyte ratio (PLR) could serve as a predictive biomarker for severe toxicities.

**Methods:**

In this retrospective study, 15 patients with relapsed or refractory DLBCL treated with tisagenlecleucel were analyzed. PLR was measured prior to lymphodepletion. Receiver operating characteristic (ROC) analysis was performed to determine the optimal PLR cutoff for predicting severe immune-related adverse events.

**Results:**

The optimal PLR cutoff was 198, with a sensitivity of 85.7% and a specificity of 87.5%. Patients with higher baseline PLR values had a significantly higher incidence of severe adverse events compared with those with lower PLR values (85.7% vs. 12.5%). Baseline PLR was positively correlated with post-infusion increases in serum ferritin, a surrogate marker of systemic inflammation. Compared with other inflammatory biomarkers, PLR demonstrated an intermediate sensitivity–specificity trade-off.

**Conclusion:**

Baseline PLR may represent a simple and accessible biomarker associated with an increased risk of severe immune-related toxicities following CAR T-cell therapy. These findings support its potential role in pre-infusion risk stratification, although validation in larger cohorts is warranted.

## 1 Introduction

The advent of anti-CD19 chimeric antigen receptor (CAR) T-cell therapy has transformed the treatment landscape for patients with relapsed or refractory diffuse large B-cell lymphoma (R/R DLBCL) (1, 2). This innovative approach offers the possibility of durable remissions in a subset of patients who have failed conventional therapies (3). However, CAR T-cell therapy is often accompanied by serious and potentially life-threatening toxicities, collectively referred to as immune effector cell-associated adverse events (IEC-AEs), which include cytokine release syndrome (CRS) and immune effector cell-associated neurotoxicity syndrome (ICANS) (4, 5). These toxicities are largely driven by systemic immune activation and cytokine-mediated inflammation, posing substantial challenges during the post-infusion period.

Although most low-grade IEC-AEs are manageable with corticosteroids or interleukin-6 inhibitors such as tocilizumab, severe (grade ≥3) toxicities remain a major cause of treatment-related morbidity and non-relapse mortality (NRM) (6). Previous studies have reported the incidence of NRM up to 5%, with many affected patients requiring intensive care support including vasopressors, mechanical ventilation, or renal replacement therapy (7, 8). Therefore, early identification of patients at high risk for severe IEC-AEs is essential for guiding preemptive monitoring strategies and timely interventions. Despite improvement in toxicity grading systems and supportive care protocols, there remains a need for reliable, easily accessible biomarkers capable of predicting severe toxicities prior to CAR T-cell infusion.

The platelet-to-lymphocyte ratio (PLR), derived from routine complete blood count (CBC) testing, has been associated with clinical outcomes in various malignancies and inflammatory conditions (9–11). PLR is thought to reflect the balance between thrombopoietic activity and immune competence, thereby serving as a surrogate marker of baseline inflammatory status. In other cytokine-mediated inflammatory settings, including COVID-19 infection, PLR has been reported to correlate with disease severity and adverse outcomes (12). These observations raise the possibility that baseline PLR may capture an underlying inflammatory vulnerability relevant to CAR T-cell–associated toxicities; however, its role in this specific clinical context has not been systematically evaluated.

In this retrospective study, we investigated whether baseline PLR, measured prior to lymphodepletion, is associated with the development of severe IEC-AEs in patients with R/R DLBCL undergoing anti-CD19 CAR T-cell therapy.

## 2 Methods

### 2.1 Study design and patients

This retrospective study included 15 consecutive patients with relapsed or refractory diffuse large B-cell lymphoma, not otherwise specified (DLBCL, NOS) who received anti-CD19 CAR T-cell therapy with tisagenlecleucel at Korea University Anam Hospital between November 2023 and April 2025. In all patients, complete blood count and serum ferritin data were available prior to the initiation of lymphodepletion. Clinical data, laboratory values, and data on IEC-AEs were retrospectively collected from medical records.

The primary objective of the study was to assess whether the baseline PLR could predict the development of severe IEC-AEs, defined as grade ≥3 CRS or ICANS. Secondary objectives included the evaluation of additional inflammatory biomarkers - such as the neutrophil-to-lymphocyte ratio (NLR), C-reactive protein (CRP), absolute lymphocyte count (ALC), and serum ferritin- for their potential associations with IEC-AEs. The relationship between these biomarkers and systemic inflammatory responses was also explored, with systemic inflammation quantified by the fold change in serum ferritin levels before and after the CAR T-cell infusion.

### 2.2 Treatment and assessment of IEC-AEs

All patients received lymphodepletion chemotherapy consisting of fludarabine (30 mg/m²/day) and cyclophosphamide (300 mg/m²/day) for three consecutive days, followed by a single infusion of tisagenlecleucel. Bridging therapy was permitted at the discretion of the treating physician based on disease status and tumor burden prior to lymphodepletion. Therapeutic modalities used for bridging included systemic chemotherapy, and radiotherapy (RT).

All IEC-AEs, including CRS and ICANS, were graded according to the American Society for Transplantation and Cellular Therapy (ASTCT) consensus criteria (13). Supportive interventions - including administration of tocilizumab and/or corticosteroids, intensive care unit (ICU) admission, and continuous renal replacement therapy (CRRT) - were documented for all study subjects. At our institution, CRS and ICANS were managed according to ASTCT-based consensus guidelines. Tocilizumab was initiated for grade ≥2 CRS or persistent fever with hemodynamic compromise, while corticosteroids were administered for grade ≥3 CRS or any grade of ICANS. ICU admission was considered for patients requiring vasopressor support, high-flow oxygen therapy, or those with rapidly progressive neurotoxicity.

### 2.3 Laboratory assessments

Baseline laboratory values were defined as those obtained on the day of or immediately prior to the initiation of lymphodepletion chemotherapy. These included CBC, serum ferritin, and CRP. The PLR was calculated by dividing the absolute platelet count by the absolute lymphocyte count from the same sample; the NLR was derived similarly. The ferritin fold change was calculated as the ratio of the highest ferritin level observed during the CAR T-cell therapy course to the baseline ferritin level prior to lymphodepletion.

Because metabolic tumor volume (MTV) was not routinely available, baseline disease burden was approximated using serum lactate dehydrogenase (LDH), a routinely measured marker in DLBCL. To identify candidate biomarkers associated with severe IEC-AEs, baseline inflammatory and hematologic markers were compared between patients who did and did not develop severe events. Variables analyzed included PLR, NLR, CRP, serum ferritin, ALC, absolute neutrophil count (ANC), platelet count. In addition, composite risk scores such as CAR-HEMATOTOX (14) and Endothelial Activation and Stress Index (EASIX) (15), as well as modified EASIX (m-EASIX) (16), were also evaluated for their associations with severe IEC-AEs.

### 2.4 Statistical analysis

Comparisons of continuous variables (e.g., PLR and serum ferritin) between patients with and without severe IEC-AEs were performed using the Mann–Whitney U test, given the non-parametric distribution of the data. Receiver operating characteristic (ROC) curve analyses were conducted to evaluate the discriminative performance of individual biomarkers, including PLR, C-reactive protein (CRP), and absolute lymphocyte count (ALC), for severe IEC-AEs. The area under the curve (AUC) and corresponding 95% confidence intervals (CI) were calculated for each marker. Optimal cutoff values were determined by maximizing the Youden index (sensitivity + specificity – 1). Based on the results of ROC analysis, PLR was selected for dichotomization, and patients were stratified into PLR-high and PLR-low groups according to the ROC-derived threshold. Differences in the incidence of severe IEC-AEs between groups were compared using Fisher’s exact test. To explore the association between baseline PLR and systemic inflammatory response following CAR T-cell infusion, correlation analyses were performed using logarithmically transformed baseline PLR and ferritin fold change (peak/baseline). Spearman’s rank correlation coefficient was used for the primary analysis. Sensitivity analyses were additionally conducted after exclusion of influential outliers defined by Cook’s distance, and correlations were reassessed using Pearson’s correlation coefficient.

All statistical analyses and visualizations were performed using R software (version 4.3.2; R Foundation for Statistical Computing, Vienna, Austria). A two-sided *p*-value of less than 0.05 was considered statistically significant for all tests.

## 3 Results

### 3.1 Patient characteristics

A total of 15 patients with relapsed or refractory diffuse large B-cell lymphoma treated with tisagenlecleucel were included in the analysis (**Table 1**). The median age was 65 years (range, 26–76), and 11 patients (73.3%) were male. All patients had an Eastern Cooperative Oncology Group (ECOG) performance status of 0 or 1. Most patients had received two prior lines of therapy (73.3%), while three patients (20.0%) had received three lines and one patient (6.7%) had received four or more. One patient (6.7%) had previously undergone autologous stem cell transplantation. All patients received bridging therapy prior to lymphodepletion. Among the 12 patients who received systemic chemotherapy as bridging therapy, 9 were treated with polatuzumab vedotin plus bendamustine and rituximab (pola-BR), 2 with bendamustine and rituximab (BR), and 1 with ifosfamide, carboplatin, and etoposide (ICE) (**Supplementary Table 1**). The remaining 3 patients received radiotherapy as bridging therapy. Post-bridging PET-CT assessment prior to lymphodepletion was not uniformly available.

**Table 1:** Baseline characteristics of patients.

Characteristics	Total patients (n=15)	PLR-High (n=7)	PLR-Low (n=8)	*p*-value
Age, y				
Median (range)	65 (26–76)	64	57	0.321
Sex, n (%)				1.000
Mal	11 (73.3%)	5 (71.4%)	6 (75.0%)	
Femal	4 (26.7%)	2 (28.6%)	2 (25.0%)	
ECOG PS				1.000
0	1 (6.7%)	0 (0.0%)	1 (12.5%)	
1	14 (93.3%)	7 (100.0%)	7 (87.5%)	
Previous Line, n (%)				0.506
2	11 (73.3%)	6 (85.7%)	5 (62.5%)	
3	3 (20.0%)	1 (14.3%)	2 (25.0%)	
4	1 (6.7%)	0 (0.0%)	1 (12.5%)	
ASCT, n (%)				1.000
Yes	1 (6.7%)	0 (0.0%)	1 (12.5%)	
No	14 (93.3%)	7 (100.0%)	7 (87.5%)	
Bridge chemotherapy, n (%)				0.092
Bendamustine-based chemotherapy	11 (73.3%)	4 (57.1%)	7 (87.5%)	
Other systemic chemotherapy	1 (6.7%)	0 (0.0%)	1 (12.5%)	
Radiotherapy	3 (20.0%)	3 (42.9%)	0 (0.0%)	
LDH (U/L)				0.867
Median (IQR)	289 (222.5-421)	281.0 (194.5 - 462.5)	292.5 (246.0 - 368.0)	
Platelet count (×10³/µL)				0.336
Median (IQR)	92 (49.5 - 184.5)	140 (67.5 - 187)	73 (37.5 - 172.5)	
Absolute lymphocyte count (/µL)				0.009
Median (IQR)	603 (245.5 - 944.5)	231.0 (176.5 - 431.9)	880 (606.8 - 2473.2)	
C-reactive protein (mg/L)				0.336
Median (IQR)	5.1 (2.4 - 35.8)	31.4 (3.9 - 40.2)	4.1 (1.4 - 28.2)	
Ferritin (ng/mL)				0.463
Median (IQR)	893.1 (549 - 2642.2)	856.8 (437.9 -1745.2)	1774.0 (635.6 - 2690.4)	

PLR, platelet-to-lymphocyte ratio; ECOG PS, Eastern Cooperative Oncology Group performance status; ASCT, autologous stem cell transplantation; LDH, lactate dehydrogenase; IQR, interquartile range.

Baseline laboratory parameters showed substantial interpatient variability (**Table 1** and **Supplementary Table 2**). Median values of key hematologic and inflammatory markers included an absolute lymphocyte count of 603/µL (interquartile range [IQR], 245.5–944.5), a platelet count of 92 ×10³/µL (IQR, 49.5–184.5), a serum ferritin level of 893.1 ng/mL (IQR, 549–2642.2), and a lactate dehydrogenase (LDH) level of 289 U/L (IQR, 222.5–421). Composite inflammatory risk scores also demonstrated wide distributions across the cohort, with median values of 5.0 (IQR, 1.5–5.0) for CAR-HEMATOTOX, 3.4 (IQR, 1.2–5.6) for EASIX, and 17.7 (IQR, 2.4–271.2) for m-EASIX.

### 3.2 Incidence and severity of IEC-AEs

Overall treatment responses were assessed descriptively. Best overall response following CAR T-cell therapy was progressive disease in 7 patients (46.7%), complete response in 5 patients (33.3%), and not evaluable in 3 patients (20.0%).

CRS of any grade occurred in 13 patients (86.7%) (**Table 2**), among whom four (26.7%) experienced severe CRS of grade 3 or 4. ICANS occurred in 5 patients (33.3%), including 3 patients (20.0%) who developed grade 4 neurotoxicity. Overall, 6 patients (40.0%) experienced at least one IEC-AE of grade 3 or higher.

**Table 2:** Incidence and severity of immune effector cell-associated adverse events (IEC-AEs).

Variable	N (%) (n=15)
CRS	
None	2 (13.3%)
Grade 1	7 (46.7%)
Grade 2	2 (13.3%)
Grade 3	3 (20.0%)
Grade 4	1 (6.7%)
ICANS	
None	10 (66.7%)
Grade 3	2 (13.3%)
Grade 4	3 (20.0%)
Tocilizumab administered	
No	8 (53.3%)
Yes	7 (46.7%)
Steroid administered	
No	10 (66.7%)
Yes	5 (33.3%)
ICU care required	
No	10 (66.7%)
Yes	5 (33.3%)
CRRT required	
No	12 (80.0%)
Yes	3 (20.0%)

CRS, cytokine release syndrome; ICANS, immune effector cell–associated neurotoxicity syndrome; ICU, intensive care unit; CRRT, continuous renal replacement therapy.

To manage these toxicities, tocilizumab was administered to 7 patients (46.7%), and corticosteroids were administered to 5 patients (33.3%). Additionally, 5 patients (33.3%) required admission to an intensive care unit (ICU), and CRRT was initiated for three patients (20%) due to severe complications.

### 3.3 Baseline inflammatory markers and predictive performance

LDH levels, used as a surrogate marker of disease burden, did not differ significantly between patients with and without severe IEC-AEs (median 287 vs 389 U/L, p=0.536). Other inflammatory markers were compared between the two groups (**Table 3**). Patients who developed severe events had significantly higher CRP levels (median 36.6 vs. 2.42 mg/L, p = 0.014) and lower absolute lymphocyte counts (median 231 vs. 880/µL, p = 0.0289). No statistically significant differences were observed in NLR, serum ferritin, platelet count, or composite inflammatory risk scores, including CAR-HEMATOTOX, EASIX, and m-EASIX.

**Table 3:** Baseline laboratory and inflammatory marker comparison and ROC summary according to severe IEC-AEs.

Variable	Non-severe (n=8), Median	Severe (n=7), Median	*p*-value	ROC AUC	Cut-off	Sensitivity	Specificity
PLR	156	342	0.0401	0.82	198	0.8571	0.875
CRP	2.42	36.6	0.014	0.88	3.1	1	0.625
ALC	880	231	0.0289	0.84	245.5	0.5714	1
NLR	2.86	8.20	0.189				
Ferritin	1034	893	0.855				
ANC	2118	1800	0.779				
Platelet	117500	79000	1				
LDH	287	389	0.536				
CAR-HEMATOTOX	4.5	5	0.903				
EASIX	3.18	3.44	1				
m-EASIX	9.86	237	0.121				

ROC, receiver operating characteristic; AUC, area under the curve; PLR, platelet-to-lymphocyte ratio; CRP, C-reactive protein; ALC, absolute lymphocyte count; NLR, neutrophil-to-lymphocyte count; ANC, absolute neutrophil count; LDH, lactate dehydrogenase; EASIX, endothelial activation and stress index; m-EASIX, modified endothelial activation and stress index.

**Figure 1:**
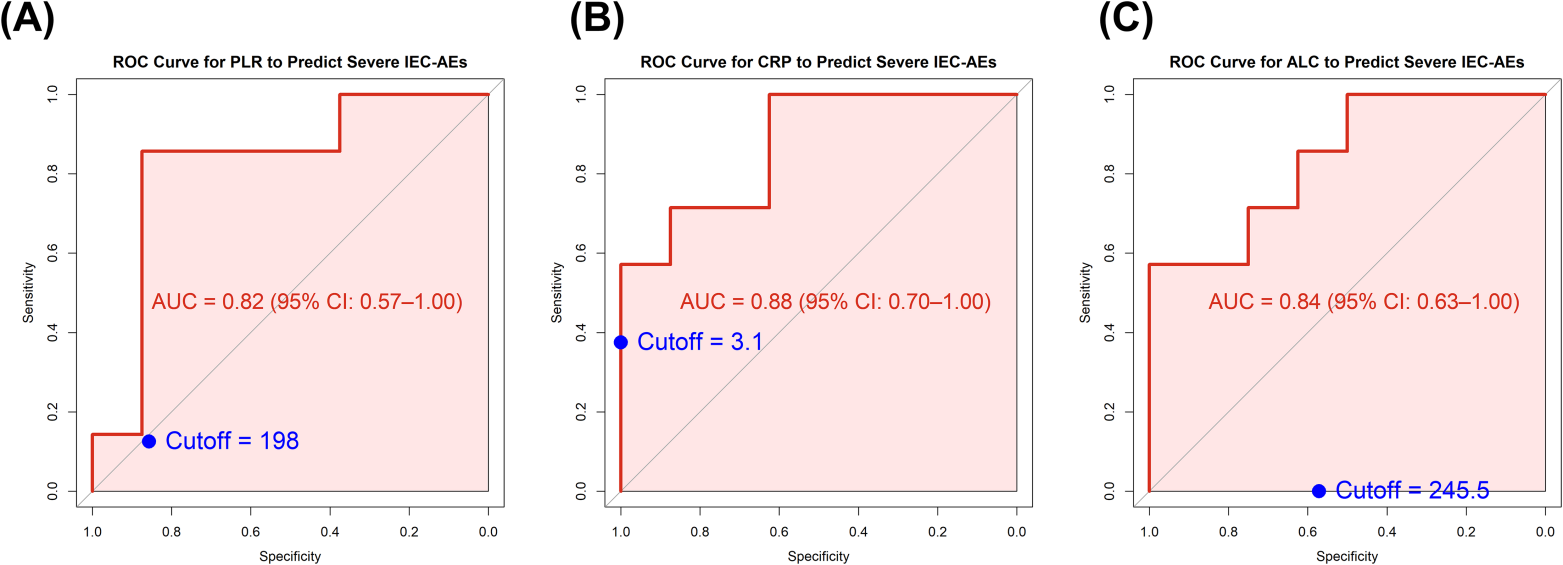
ROC curve for baseline inflammatory marker in predicting severe IEC-AEs. **(A)** Platelet-to-lymphocyte ratio (PLR). **(B)** C-reactive protein (CRP). **(C)** absolute lymphocyte count (ALC).

To further evaluate the predictive performance of these biomarkers, ROC curve analyses were performed. Among the tested variables, PLR demonstrated a sensitivity–specificity profile that was intermediate between CRP and ALC, with an AUC of 0.82 (95% CI: 0.57–1.00) (**Figure 1A**). The optimal cutoff value for PLR, determined by maximizing Youden’s index, was 198, yielding a sensitivity of 85.7% and a specificity of 87.5%. CRP showed the highest AUC at 0.88 (95% CI: 0.70–1.00), achieving 100% sensitivity at a cutoff value of 3.1 mg/L; however, its specificity was lower (62.5%) (**Figure 1B**). ALC showed an AUC of 0.84 (95% CI: 0.63–1.00), with a high specificity of 100% at a cutoff value of 245.5 µL, but lower sensitivity (57.1%) (**Figure 1C**).

Collectively, PLR, CRP, and ALC showed discriminatory potential for severe IEC-AEs, with PLR providing an intermediate sensitivity–specificity trade-off between CRP (higher sensitivity, lower specificity) and ALC (higher specificity, lower sensitivity) in this exploratory cohort.

### 3.4 Association between PLR stratification and IEC-AEs incidence

**Figure 2:**
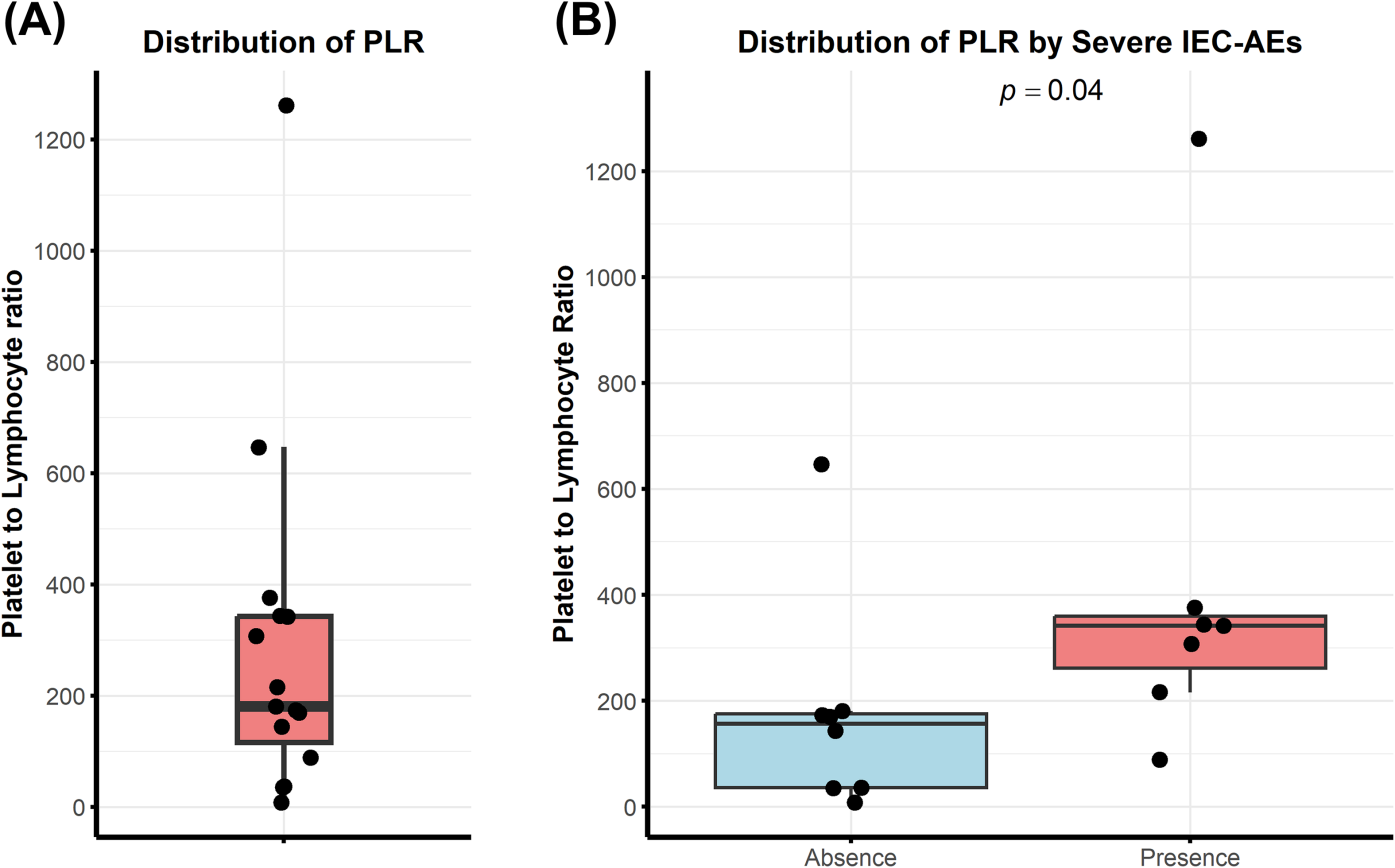
Distribution of baseline PLR and its association with severe IEC-AEs. **(A)** Overall distribution of baseline platelet-to-lymphocyte ratio **(PLR)** in the study cohort. **(B)** Comparison of baseline PLR values between patients with and without severe IEC-AEs.

Baseline PLR values demonstrated substantial interpatient variability in the overall cohort, with a median value of 180.4 (**Figure 2A**). When patients were stratified according to the occurrence of severe IEC-AEs, those who developed grade ≥3 CRS or ICANS had significantly higher baseline PLR values than those without severe events (median, 342 vs. 156; p = 0.0401; **Figure 2B**).

**Figure 3:**
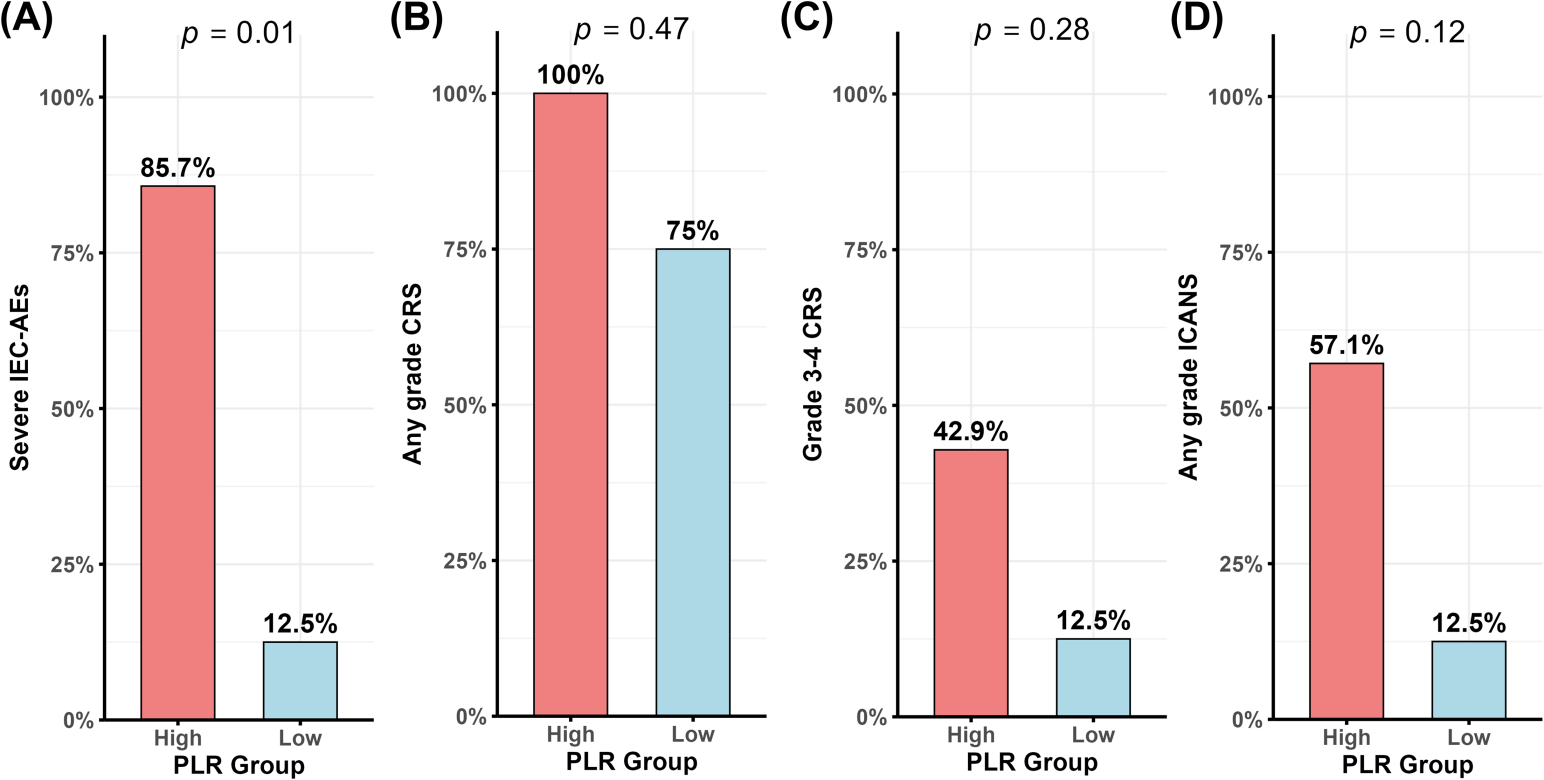
Incidence of IEC-AEs according to baseline PLR. **(A)** Severe IEC-AEs (Grade ≥3 CRS or ICANS). **(B)** Any grade CRS. **(C)** Grade ≥3 CRS. **(D)** Any grade ICANS.

Based on the ROC-derived optimal cutoff value of 198, patients were further categorized into PLR-high and PLR-low groups. The incidence of severe IEC-AEs (grade ≥3 CRS or ICANS) was significantly higher in the PLR-high group compared with the PLR-low group (85.7% vs. 12.5%; p = 0.01; **Figure 3A**). Although differences in individual adverse event categories did not reach statistical significance, higher frequencies of CRS and ICANS were consistently observed in the PLR-high group, including any grade CRS (100% vs. 75%; p = 0.47; **Figure 3B**), grade 3–4 CRS (42.9% vs. 12.5%; p = 0.28; **Figure 3C**), and any grade ICANS (57.1% vs. 12.5%; p = 0.12; **Figure 3D**).

Given the limited sample size, comprehensive multivariable modeling was not feasible. However, to partially account for potential confounding by baseline patient characteristics and disease burden, exploratory minimally adjusted logistic regression analyses were performed to assess the robustness of the association between baseline PLR and severe IEC-AEs. Across models adjusted for age and/or LDH, PLR showed a consistent direction of association; however, statistical significance was not reached (**Supplementary Table 3**).

### 3.5 Association between PLR and systemic inflammatory response

**Figure 4:**
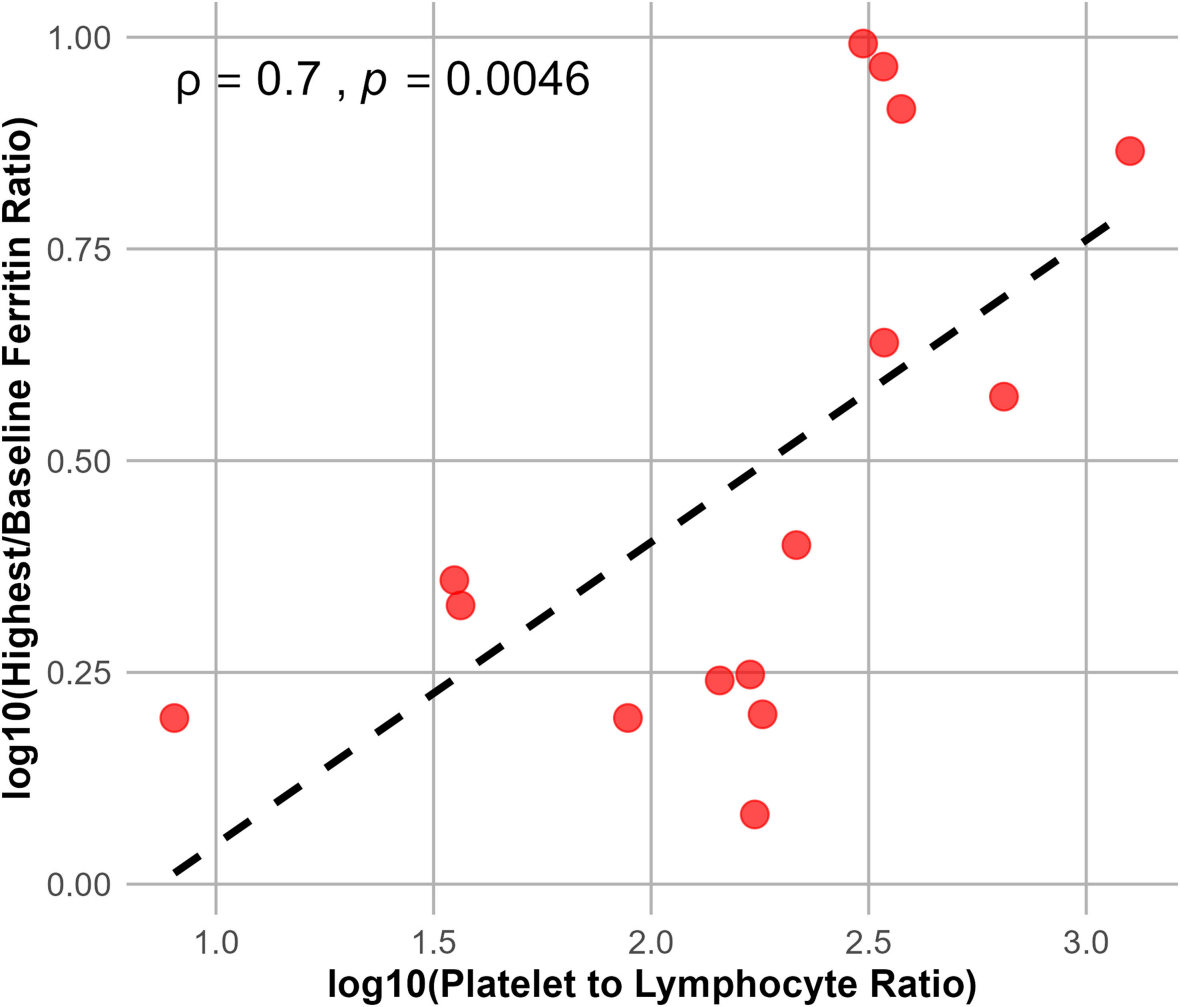
Correlation between baseline PLR and ferritin fold change after CAR T-cell therapy. Each dot represents a patient. The y-axis shows ferritin fold-change from baseline to peak, and the x-axis indicates baseline PLR before CAR T-cell infusion. Correlation was assessed using logarithmically transformed values (Spearman’s *ρ* = 0.7, p = 0.0046). The dashed line represents a fitted linear trend for visualization only.

The association between baseline inflammatory status and post-infusion systemic inflammatory response was assessed by evaluating the correlation between baseline PLR and ferritin fold change, defined as the ratio of peak serum ferritin level after CAR T-cell infusion to the baseline level prior to lymphodepletion. Correlation analyses were performed using logarithmically transformed values. Baseline PLR was positively correlated with ferritin fold change (Spearman’s ρ = 0.70, p = 0.0046; **Figure 4**).

To assess the robustness of this association, additional sensitivity analyses were conducted after exclusion of influential outliers. The direction of the correlation remained consistent in these analyses, and the association remained statistically significant using Pearson’s correlation analysis (r = 0.62, p = 0.018; **Supplementary Figure 1**).

## 4 Discussion

In this study, we observed a statistical association between higher baseline platelet-to-lymphocyte ratio (PLR) and the occurrence of severe immune effector cell–associated adverse events (IEC-AEs) among patients with R/R DLBCL undergoing anti-CD19 CAR T-cell therapy. Patients with a pre-infusion PLR ≥198 experienced a higher incidence of grade ≥3 cytokine release syndrome or immune effector cell–associated neurotoxicity syndrome compared with those with lower PLR values. Among the inflammatory biomarkers evaluated, PLR achieved an AUC of 0.82 at a threshold of 198, demonstrating clinically practical discrimination by balancing sensitivity and specificity within a single, readily available parameter. Although confidence intervals overlapped across biomarkers, these findings support its potential utility as an integrative inflammatory marker. Other inflammatory markers, including neutrophil-to-lymphocyte ratio (NLR) and baseline ferritin, as well as composite risk indices such as CAR-HEMATOTOX (14), EASIX (15) and m-EASIX (16), showed limited discriminatory capacity in this cohort. Notably, EASIX has previously shown predictive value in patients with acute lymphoblastic leukemia and multiple myeloma undergoing CAR T-cell therapy (17, 18). However, its limited performance in this DLBCL-only cohort suggests that the utility of composite endothelial stress-based scores may vary across disease subtypes, underscoring the importance of disease-specific biomarker validation. Because baseline LDH levels and other key laboratory parameters incorporated into CAR-HEMATOTOX and EASIX were comparable between severe and non-severe groups, the discriminatory capacity of these composite scores may have been attenuated in this cohort.

In addition, baseline PLR was positively associated with post-infusion ferritin fold change, used here as a dynamic surrogate of systemic inflammatory amplification following CAR T-cell therapy (Spearman’s ρ = 0.7, p = 0.0046). This finding supports the concept that a heightened pre-infusion inflammatory milieu may reflect an association between pre-infusion inflammatory status and subsequent inflammatory amplification. Although post-infusion ferritin levels are influenced by therapeutic interventions such as tocilizumab and corticosteroids (19, 20), the observed correlation likely reflects the overall magnitude of inflammatory activation rather than a direct causal relationship. Sensitivity analyses using log-transformed values yielded consistent results, supporting the robustness of this exploratory observation.

Although absolute lymphocyte count alone demonstrated high specificity for severe IEC-AEs, PLR extends beyond lymphopenia by incorporating platelet dynamics that may reflect cytokine-driven inflammatory activation. In our cohort, platelet count alone did not discriminate severe events, yet the combined ratio demonstrated predictive performance, suggesting that PLR captures a broader inflammatory and immune context rather than simply reflecting inverse lymphocyte count. Biologically, platelets are increasingly recognized as active mediators of inflammation, with thrombopoiesis upregulated by cytokines such as IL-6, IL-1β, and TNF-α, all implicated in the pathogenesis of CRS and ICANS (21–23). Simultaneously, lymphopenia is associated with immune dysregulation, T-cell exhaustion, and treatment-induced immunosenescence (24–26), impairing regulatory capacity and enhancing susceptibility to immune-mediated toxicity. Elevated PLR therefore integrates signals of inflammatory amplification and reduced regulatory capacity, aligning closely with mechanisms underlying severe CAR T-cell–associated toxicities.

The relevance of PLR as a hyperinflammatory marker is further supported by findings from other cytokine-mediated inflammatory conditions. During the COVID-19 pandemic, PLR emerged as a reliable biomarker of disease severity and cytokine storm, with several meta-analyses identifying threshold values between 180 and 210 that were predictive of critical illness and mortality. For example, Simon et al. identified a PLR greater than 200 as a predictor of mortality in patients presenting to the emergency department with a diagnosis of SARS-CoV-2 infection (27), and Sarkar et al. reported an association between PLR greater than 180 and mortality in a pooled analysis (12). Notably, the PLR cutoff 198 associated with severe IEC-AEs in our cohort falls within the range reported in other cytokine-mediated inflammatory conditions, such as COVID-19, suggesting that PLR may reflect a shared inflammatory susceptibility. Although the initiating triggers differ between CAR T-cell–associated toxicities and COVID-19, both syndromes are characterized by dysregulated cytokine amplification. The convergence of PLR thresholds across these distinct inflammatory contexts reinforces the biological plausibility of PLR as a marker of systemic inflammatory susceptibility.

Several limitations warrant consideration. The small sample size and retrospective single-center design limit statistical power and preclude comprehensive multivariable adjustment. Detailed metabolic tumor volume data were unavailable, and baseline disease burden was approximated using serum LDH, an imperfect surrogate in aggressive lymphomas. Although bridging modality differed between PLR strata, this likely reflected underlying disease characteristics rather than a direct effect on toxicity risk; nevertheless, residual confounding cannot be excluded. Finally, the present findings are specific to patients treated with tisagenlecleucel, and their generalizability to other CAR T-cell products or disease entities remains uncertain (28). Accordingly, the identified PLR cutoff should be considered hypothesis-generating, and prospective validation in larger, multicenter cohorts is warranted to clarify the clinical relevance of PLR and its potential role in pre-infusion risk assessment frameworks.

In summary, baseline PLR, a simple and readily accessible laboratory parameter, was associated with severe IEC-AEs and may serve as an integrative marker of inflammatory vulnerability prior to CAR T-cell infusion. If validated in larger cohorts, PLR could represent a practical addition to pre-infusion toxicity risk assessment frameworks.

## Data Availability

The original contributions presented in the study are included in the article/**Supplementary Material**. Further inquiries can be directed to the corresponding author.
