# Supplementary figures and images for: Baseline platelet-to-lymphocyte ratio is associated with severe immune effector cell-associated toxicities in diffuse large B-cell lymphoma patients receiving anti-CD19 CAR T-cell therapy
Source: Front Immunol. 2026 Mar 24;17:1731711. doi: 10.3389/fimmu.2026.1731711 (PMC13055538; doi:10.3389/fimmu.2026.1731711)

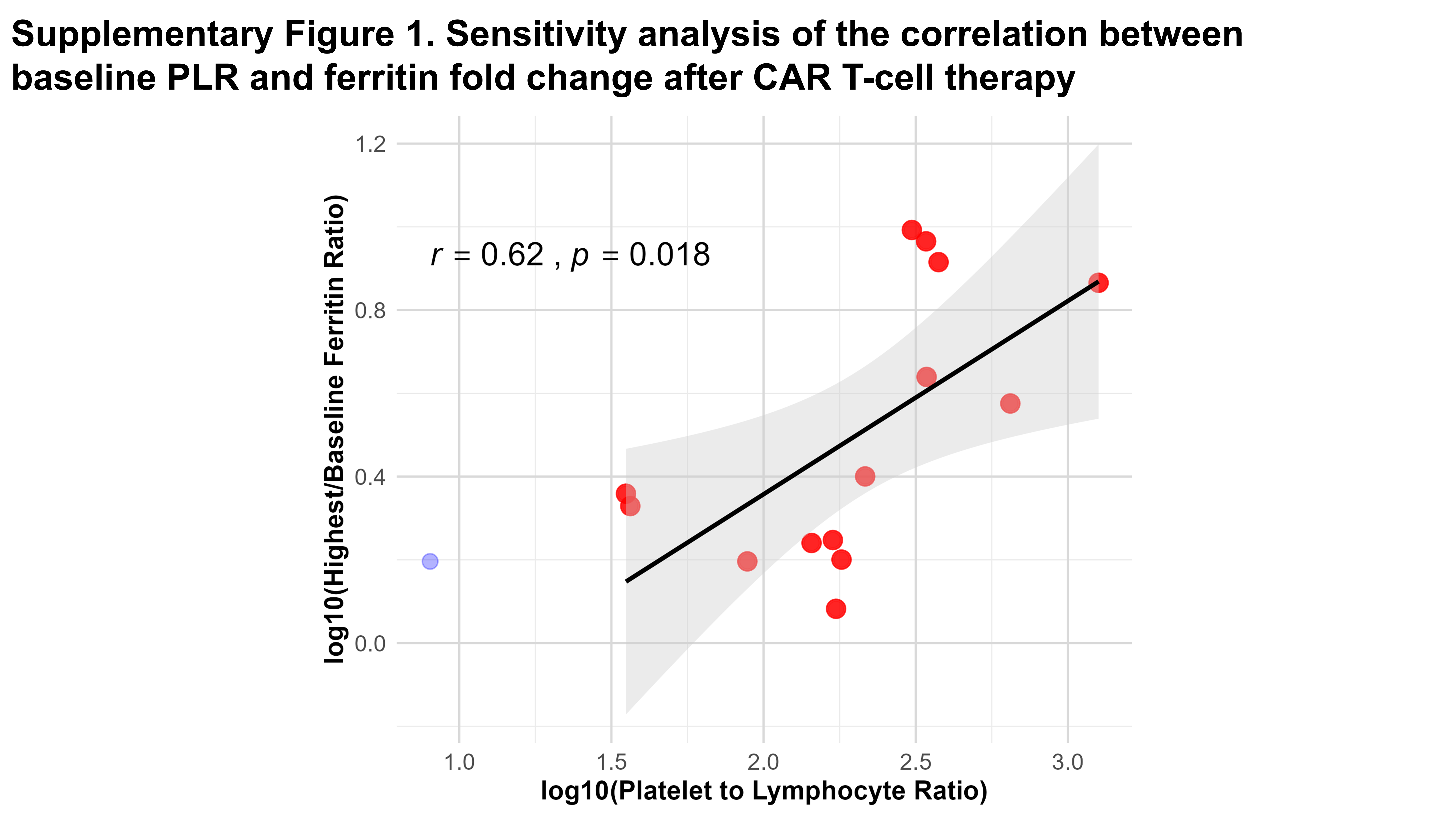

Supplement: Supplementary file 1 [file Image1.tif]
