# Supplementary material for: Baseline platelet-to-lymphocyte ratio is associated with severe immune effector cell-associated toxicities in diffuse large B-cell lymphoma patients receiving anti-CD19 CAR T-cell therapy
Source: Front Immunol. 2026 Mar 24;17:1731711. doi: 10.3389/fimmu.2026.1731711 (PMC13055538; doi:10.3389/fimmu.2026.1731711)
Supplement: Supplementary file 2 [file Table1.docx]

| Characteristics | Total patients  (n=15) | PLR-High  (n=7) | PLR-Low  (n=8) | *p*-value |
| --- | --- | --- | --- | --- |
| Age, y  Median (range) | 65 (26–76) | 64 | 57 | 0.321 |
| Sex, n (%)  Male  Female | 11 (73.3%)  4 (26.7%) | 5 (71.4%)  2 (28.6%) | 6 (75.0%)  2 (25.0%) | 1.000 |
| ECOG PS  0  1 | 1 (6.7%)  14 (93.3%) | 0 (0.0%)  7 (100.0%) | 1 (12.5%)  7 (87.5%) | 1.000 |
| Previous Line, n (%)  2  3  4 | 11 (73.3%)  3 (20.0%)  1 (6.7%) | 6 (85.7%)  1 (14.3%)  0 (0.0%) | 5 (62.5%)  2 (25.0%)  1 (12.5%) | 0.506 |
| ASCT, n (%)  Yes  No | 1 (6.7%)  14 (93.3%) | 0 (0.0%)  7 (100.0%) | 1 (12.5%)  7 (87.5%) | 1.000 |
| Bridge chemotherapy, n (%)  Bendamustine-based chemotherapy  Other systemic chemotherapy  Radiotherapy | 11 (73.3%)  1 (6.7%)  3 (20.0%) | 4 (57.1%)  0 (0.0%)  3 (42.9%) | 7 (87.5%)  1 (12.5%)  0 (0.0%) | 0.092 |
| LDH (U/L)  Median (IQR) | 289 (222.5-421) | 281.0 (194.5 - 462.5) | 292.5 (246.0 - 368.0) | 0.867 |
| Platelet count (×10³/µL)  Median (IQR) | 92 (49.5 - 184.5) | 140 (67.5 - 187) | 73 (37.5 - 172.5) | 0.336 |
| Absolute lymphocyte count (/µL)  Median (IQR) | 603 (245.5 - 944.5) | 231.0 (176.5 - 431.9) | 880 (606.8 - 2473.2) | 0.009 |
| C-reactive protein (mg/L)  Median (IQR) | 5.1 (2.4 - 35.8) | 31.4 (3.9 - 40.2) | 4.1 (1.4 - 28.2) | 0.336 |
| Ferritin (ng/mL)  Median (IQR) | 893.1 (549 - 2642.2) | 856.8 (437.9 -1745.2) | 1774.0 (635.6 - 2690.4) | 0.463 |

**Supplementary Table 1** Bridging therapy modalities and post-bridging disease status prior to lymphodepletion

|  | PLR-High  (n=7) | PLR-Low  (n=8) | *p*-value |
| --- | --- | --- | --- |
| Bridge chemotherapy, n (%)  Pola-BR  BR  ICE  Radiotherapy | 2 (28.6%)  2 (28.6%)  0 (0.0%)  3 (42.9%) | 7 (87.5%)  0 (0.0%)  1 (12.5%)  0 (0.0%) | 0.033 |
| PET response after bridging  CR  PR  PD  Not available^a^ | 0 (0.0%)  0 (0.0%)  3 (42.9%)  4 (57.1%) | 2 (25.0%)  1 (12.5%)  2 (25.0%)  3 (37.5%) | 0.349 |

^a^Post-bridging PET-CT assessment prior to lymphodepletion was available in a subset of patients, as it was introduced during the study period.

Abbreviations: Pola-BR, polatuzumab vedotin + bendamustine + rituximab; BR, bendamustine + rituximab; ICE, ifosfamide + carboplatin + etoposide; RT, radiotherapy; CR, complete response; PR, partial response; PD, progressive disease.

**Supplementary Table 2** Overall distribution of baseline laboratory parameters and composite inflammatory scores

| Laboratory finding | Median [Interquartile range] |
| --- | --- |
| Platelet to lymphocyte ratio  Neutrophil to lymphocyte ratio  Hemoglobin (g/dL)  White blood cell count (/µL)  Absolute neutrophil count (/µL)  Creatinine (mg/dL)  Total Bilirubin (mg/dL)  CAR-HEMATOTOX  EASIX  m-EASIX | 180.4 [116.1 - 342.6]  3.2 [2.4 - 10.1]  9.5 [8.6 - 10.5]  2590 [2080 - 5175]  1800 [1238.8 - 2857.5]  0.8 [0.7 – 1.0]  0.6 [0.4 – 0.7]  5.0 [1.5 – 5.0]  3.4 [1.2 – 5.6]  17.7 [2.4 – 271.2] |

**Supplementary Table 3** Exploratory minimally adjusted logistic regression analyses of baseline PLR and severe IEC-AEs

| Model | Variable | OR (95% CI) | *p*-value |
| --- | --- | --- | --- |
| Model 1 | PLR (per SD increase) | 3.60 (0.851 – 58.2) | 0.223 |
|  | Age (per 10-year increase) | 1.25 (0.520 – 3.95) | 0.635 |
| Model 2 | PLR (per SD increase) | 3.97 (0.864 – 66.7) | 0.212 |
|  | LDH (per SD increase) | 1.03 (0.221 – 3.30) | 0.962 |
| Model 3 | PLR (per SD increase) | 3.65 (0.850 - 59.8) | 0.221 |
|  | Age (per 10-year increase) | 1.27 (0.520 – 4.17) | 0.627 |
|  | LDH (per SD increase) | 1.08 (0.219 – 3.74) | 0.899 |
